# Quantitative and Qualitative Difference in Antibody Response against Omicron and Ancestral SARS-CoV-2 after Third and Fourth Vaccination

**DOI:** 10.3390/vaccines10050796

**Published:** 2022-05-17

**Authors:** Sascha Hein, Ines Mhedhbi, Tobias Zahn, Catarina Sabino, Nuka Ivalu Benz, Younes Husria, Patricia Maria Renelt, Floriane Braun, Doris Oberle, Thorsten J. Maier, Christoph Hildt, Eberhard Hildt

**Affiliations:** 1 Department of Virology, Paul-Ehrlich-Institut, Paul-Ehrlich Street 51-59, D-63225 Langen, Germany; sascha.hein@pei.de (S.H.); ines.mhedhbi@pei.de (I.M.); tobias.zahn@pei.de (T.Z.); catarina.sabino@pei.de (C.S.); nukaivalu.benz@pei.de (N.I.B.); younes.husria@pei.de (Y.H.); patriciamaria.renelt@pei.de (P.M.R.); florianeclaudiamaria.braun@pei.de (F.B.); 2 Safety of Medicinal Products and Medical Devices Division, Paul-Ehrlich-Institut, D-63325 Langen, Germany; doris.oberle@pei.de (D.O.); thorstenjuergen.maier@pei.de (T.J.M.); 3 Main-Kinzig-Kliniken, D-63571 Gelnhausen, Germany; christoph.hildt@mkkliniken.de

**Keywords:** SARS-CoV-2, Omicron, BNT162b2, second booster, antibody response

## Abstract

Waning immunity against SARS-CoV-2 and the emergence of variants, especially of the most distant variant, Omicron, affect titers of neutralizing antibodies in the sera of vaccinated individuals. Thus, two vaccinations with the mRNA vaccine BNT162b fail to induce neutralizing antibodies against the Omicron variant. A first booster vaccination increases Omicron-RBD-binding IgG and IgA and neutralizing capacity. In comparison, the Wuhan isolate titers of the Omicron variant binding antibodies are 8.5 lower. After a third vaccination, induction of Omicron-RBD- and Wuhan-RBD-binding antibodies follows the same kinetic. Five to six months after the third vaccination, there are still Omicron-RBD-binding antibodies detectable, but 35.9 percent of the analyzed sera fail to neutralize the Omicron variant, while all sera efficiently neutralize the Delta isolate. In the case of the Wuhan-RBD, a significantly larger number of stable antigen–antibody complexes is formed than in Omicron-RBD. A fourth vaccination with mRNA-1273 temporarily restores levels of Omicron-, Delta- and Wuhan-specific antibodies. Comparing different booster strategies revealed that the breadth of the immune response is not affected by the vaccination regimen. Taken together, these data indicate that booster vaccinations (third and fourth dose) increase the breadth of the immune response, but there is a qualitative difference of antibodies with respect to the stability of antigen–antibody complexes and persistence of antibody titers.

## 1. Introduction

Since the end of 2019, the SARS-CoV-2 pandemic has rapidly spread all over the world. Based on an unprecedented effort about one year later, the first vaccines were approved and mass vaccination campaigns started. For the mRNA vaccines (mRNA-1273 and BNT162b2), real-world data revealed a very high vaccine effectiveness of more than 90% VE with respect to prevention of COVID-19 [1,2]. However, over time, a waning of the immunity was observed, especially in people above 60 years old, although the vaccine effectiveness with respect to prevention of severe disease or death was still high [3]. Moreover, the emergence of variants of concern, especially of the Omicron variant, which differs in the spike protein in 37 positions as compared to the spike protein of the Wuhan isolate, is the antigen of the licensed vaccines contributes to this [4,5]. The Omicron variant has rapidly emerged worldwide [6]. Very high transmission rates but a less pronounced virulence compared to the previous variants of concern characterize this variant [7,8]. The very high transmissibility caused a dramatic increase in cases, which bears the risk of causing a collapse of healthcare systems. The emergence of Omicron was a further reason for a third vaccination. In autumn 2021, the FDA granted a EUA (emergency use authorization) for booster doses of the BioNTech/Pfizer (BNT162b2) vaccine and for the Moderna (mRNA-1273) vaccine, which was also approved by the EMA based on a variation of the conditional market authorizations for the two vaccines.

There is an obvious difference between the ancestral-based antigen of the licensed vaccines and the Omicron variant with a spike protein that differs in 37 residues and thereby affects the binding of neutralizing antibodies. The impact of a Wuhan-spike-based vaccination on the development of Omicron-specific antibodies is not fully clear. A recent report described that pseudotyped particles with a designed spike protein that differs in 25 positions from the Wuhan-spike protein were not neutralized by sera obtained from individuals vaccinated twice or by convalescent sera [9]. However, sera from individuals with a breakthrough infection after two vaccinations or after a third vaccination developed neutralizing capacity. It is assumed that broadening the immune response by somatic hypermutation is causative for this effect. However, there is still a variety of open questions concerning the antibody response after a booster vaccination. This study aims to characterize the kinetic parameters of the increase in antibody titers against variants after booster and the waning with respect to the different variants. In addition to binding and neutralizing titers, the evolution of the affinity over time after booster vaccination to different variants is characterized.

## 2. Materials and Methods

### 2.1. Cell Culture and Virus Strains

Vero E6 (ATCC^®^ CRL-1586™) and HEK293T cells (ATCC CRL-3216™) were used for the experimental work of this study. Cells were cultivated in Dulbecco’s Modified Eagle’s medium (DMEM, Sigma, Taufkirchen, Germany) supplemented with 10% fetal bovine serum (FBS Bio & SELL GmbH, Feucht, Germany), 1% penicillin/streptomycin and 1% L-glutamine, and incubated with 5% CO_2_ at 37 °C. The SARS-CoV-2 variants of concern belonging to the Pangolin lineages B.1.1.7, B 1.351, B.1.1.617.2 and B.1.1.529 as well as an ancestral isolate UVE/SARS-CoV-2/2020/FR/702 (GenBank: MT777677.1) were used. EVAg and the Robert Koch Institut (RKI) provided the utilized virus strains. All cell culture work involving infectious SARS-CoV-2 was performed under biosafety level-3 (BSL-3) terms.

### 2.2. RBD Protein Production

The mutant *rbd* genes were cloned into pCAGGS-sRBD plasmid, which was kindly provided by Florian Krammer (Icahn School of Medicine at Mount Sinai; Amanat et al., 2020). The Delta-*rbd* gene was created via QuikChange Site-Directed Mutagenesis using PfuUltra High-Fidelity DNA Polymerase (Agilent, Santa Clara, CA, USA) according to the protocol of the manufacturer. The *Omicron*-*rbd* gene was synthesized by Thermo Fisher Scientific (GenArt, Regensburg, Germany) and cloned into the pCAGGS vector. Sequencing was performed to validate all generated plasmids. Soluble RBD protein was produced in HEK293T cells and purified via Ni-NTA Affinity chromatography as described [10].

### 2.3. Plaque Reduction Neutralization Test (PRNT50)

For the plaque reduction neutralization test (PRNT50), which was performed in duplicates, Vero E6 cells were seeded in twelve-well plates (2.5 × 10^5^ cells per well, Greiner Bio-One, Kremsmünster, Austria) and incubated with 5% CO2 at 37 °C. A twofold serial dilution (1:20 to 1:640) of each serum was incubated for 1 h at 37 °C with 80 PFU of the respective SARS-CoV-2 strains in a total volume of 120 µL FBS-free DMEM and continued according to the protocol [10].

### 2.4. ELISA

**Figure 1:**
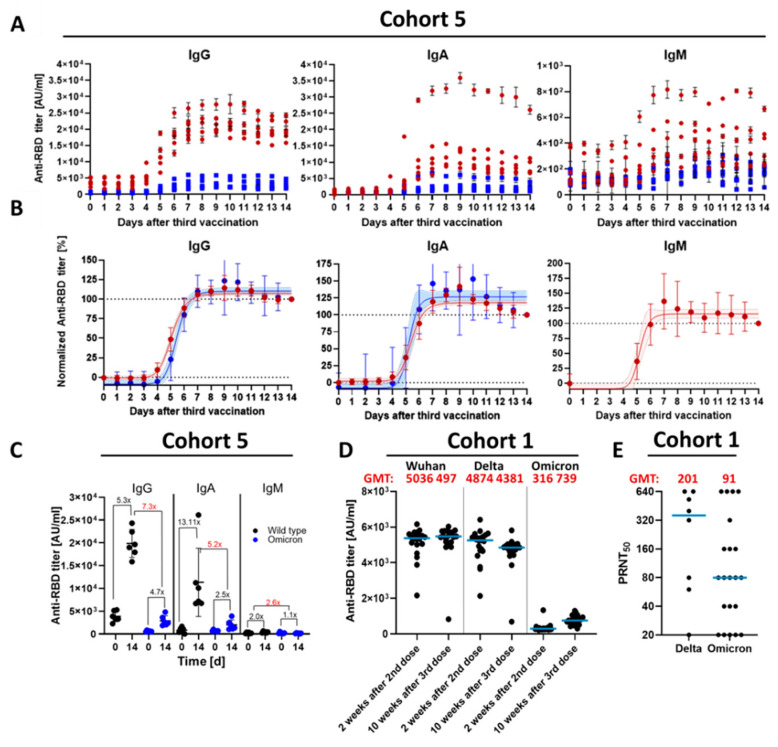
Antibody titers within 14 days after third immunization. (**A**) Absolute anti-SARS-CoV-2 IgG, IgA and IgM titers of all six subjects. Red circles: titer against Wuhan-RBD; Blue squares: titer against Omicron-RBD. (**B**) Normalized anti-SARS-CoV-2 titers. Titers from A were normalized between 0 and 100 (see Material and Methods for details). Red: titer against Wuhan-RBD; Blue: titer against Omicron-RBD; red circles: Mean values of the eight subjects with standard deviation; Line: sigmoidal 4 PL fit with 95% confidence level (dashed lines). Normalization of the anti-Omicron-RBD IgM titers was not possible. (**C**) Comparison of antibody titer before (d = 0) and two weeks after third dose (d = 14) also with respect to the variants. The time points d = 0 and d = 14 from Figure 1A were used to calculate the increase in the antibody concentration within two weeks. Black circle: ELISA Data against SARS-CoV-2 Wuhan-RBD; Blue: ELISA Data against SARS-CoV-2 Omicron-RBD. Black Numbers describe the increase in the antibody concentration over the two weeks. Red Numbers describe the decrease in the antibody titer from the Wuhan v.s. Omicron. Cut-off values for the ELISAs were: IgG: 47 AU/mL, IgM: 80 AU/mL and IgA: 6 AU/mL. (**D**) Sera from BNT162b2 (21 sera) two weeks after second dose and 10 weeks after third dose were tested by ELISA using the RBD domain from different SARS-CoV-2 variants (Delta or Omicron, Wuhan, China) as antigen. All the values were measured in duplicate and normalized against the Anti-His coating control. AU/mL; Arbitrary unit per milliliter. (**E**) Plaque reduction neutralization titer of sera from BNT162b2 10 weeks after third dose against the SARS-CoV-2 Delta and Omicron variants. Neutralization is represented by the PRNT50. In case of the Delta variant, eight sera were tested. Plaque assay was performed in duplicate. Blue bar represents the geometric mean. GMT; geometric mean titer.

Spike-RBD-specific antibodies were quantified by ELISA as described [10]. For antibody titer determination, sera were diluted 1:100 in 10% FCS in PBS and for investigating kinetics, sera (Figure 1A–C; Cohort 5) were diluted 1:500 in 10% FCS in PBS. Analysis was performed immediately at 450 nm on a Tecan reader (Tecan Group, Männedorf, Switzerland). For kinetic analysis of cohort 5 (Figure 1B), the data were normalized between 0% (day before third vaccination) and 100% (day 14 after first boost). The calculation of kinetic parameters (99% of the maximum antibody and 50% of the maximum antibody) a sigmoidal four parameter logistic (4 PL) fit with default parameters was used in Graph Pad Prism v.9.2.0.

### 2.5. Surface Plasmon Resonance Spectroscopy (SPR)

**Figure 2:**
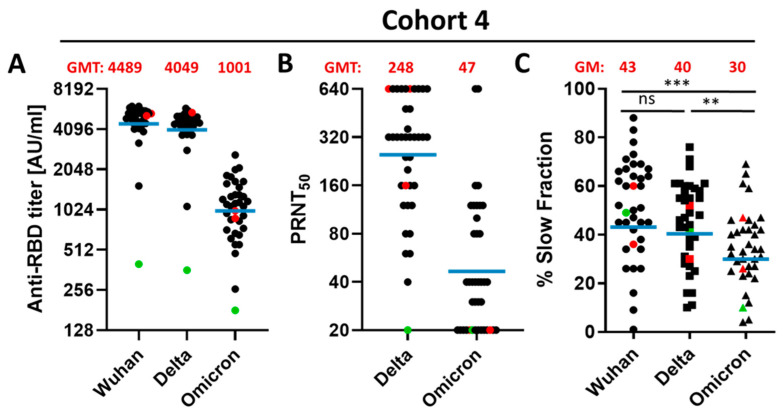
Characterization of 36 sera from patients 5–6 months after third dose of BNT162b2. (**A**) Sera were tested by ELISA using the RBD domain from different SARS-CoV-2 variants (Delta or Omicron, Wuhan, China) as antigen. AU/mL; Arbitrary unit per milliliter. (**B**) Plaque reduction neutralization titer against the SARS-CoV-2 Delta variant and the Omicron variant. (**C**) Stability of RBD-antibody complexes were analyzed by surface plasmon resonance spectroscopy (see experimental section for details). Calculated percentage of slow dissociating antibodies (slow fraction) are shown. The calculation is based on an equation describing a simultaneous dissociation of two independent populations. Blue bar represents the geometric mean. Red dots: Patients became infected with Omicron variant 3 days after blood collection. Green: Patient is immunosuppressed. GMT; geometric mean titer, GM; geometric mean. *p*-value: ***, 0.0002; **, 0.002; ns; 0.12.

**Figure 3:**
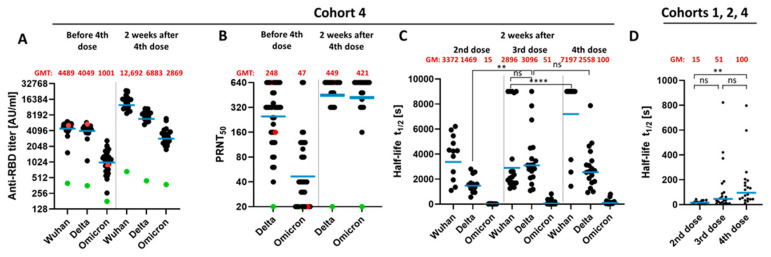
Humoral response 2 weeks after fourth dose. 19 patients received 5–6 months after the third BNT162b2 dose a fourth vaccination with 50 μg mRNA-1273. (**A**) Sera were tested by ELISA using the RBD domain from different SARS-CoV-2 variants (Delta or Omicron, Wuhan, China) as antigen. AU/mL; Arbitrary unit per milliliter. (**B**) Plaque reduction neutralization titer against the SARS-CoV-2 Delta variant and the Omicron variant. Data before the fourth dose are corresponding to the data shown in Figure 2A–B and were shown again for comparison of the fourth dose effect (**C**) Half time of RBD-antibody complexes with different RBD (Delta or Omicron, Wuhan, China) proteins were analyzed by surface plasmon resonance spectroscopy (see experimental section for details). The dissociation rate was calculated by using an equation describing a simultaneous dissociation of two independent populations. (**D**) Section of the data shown in C against Omicron. Blue bar represents the geometric mean. Red dots: Patients became infected with Omicron variant 3 days after blood collection. Green: Patient is immunosuppressed. GMT; geometric mean titer, GM; geometric mean. *p*-value: ****, <0.0001; **, 0.002; ns; 0.12.

Surface plasmon resonance spectroscopy (SPR) was carried out to characterize patient sera using the Biacore T200 system (Cytiva, Uppsala, Sweden) as described previously [11]. Due to the large number of samples and the limited lifetime of the coupled RBDs, the two experiments were performed between two chips. On chip 1 (data from Figure 2), final response levels of 419 RU for Omicron-RBD, 213 RU for Delta-RBD and 112 RU for Wuhan-RBD were obtained, while chip 2 (data from Figure 3) measured final response levels of 5007 RU for Omicron-RBD, 11051 RU for Delta-RBD and 7893 RU for Wuhan-RBD. One RU can be correlated with 1 pg RBD protein/mm^2^_._

### 2.6. Ethics and Collected Sera 

The sera of this vaccination study, PEI-SARS-CoV-2, were provided by the Paul-Ehrlich-Institut in Langen, Germany. The study was approved by the local ethics committee (Ethik-Kommission Landesärztekammer Hessen 60314 Frankfurt am Main; 2020-1664_4-evBO) and performed according to the provisions of the Declaration of Helsinki and good clinical practice guidelines in its latest revision. All participants gave their written declaration of consent. In total, 112 healthy healthcare workers were recruited in the beginning of this study. Individuals from cohorts 2–5 had no direct contact with infected individuals and work in administrative areas. All subjects were tested negative for anti-SARS-CoV-2 N antibodies, so that a previous SARS-CoV-2 infection could be excluded. A list of recruited cohorts and collected sera is shown in Table 1.

## 3. Results

### 3.1. Kinetic Parameters of Antibody Response within 14 D after Third Vaccination

Booster vaccinations were performed to restore waning titers and to exploit the broadening of the immune response over time. However, the detailed kinetics for increasing neutralizing titers after the third dose (first boost) is unknown. Moreover, it is unclear if neutralizing titers against ancestral and Omicron variants increase with different kinetics. To investigate this, sera from six completely vaccinated volunteers (3 × BNT162b2; Cohort 5, Table 1) were collected daily over 15 days, starting one day before the third dose (day 0). ELISAs for the quantification of Wuhan-RBD and Omicron-RBD-binding antibodies revealed that absolute titers of all Ig subtypes increase within two weeks (Figure 1A). The titer of Omicron-RBD-binding antibodies is for all time points lower than that of Wuhan-RBD. The number of Wuhan-RBD-binding IgG shows a 5-fold (Omicron-RBD 4.7-fold), IgM a 3-fold (Omicron-RBD n.d.) and IgA a 13-fold (Omicron-RBD 2.5-fold) increase in binding antibodies (Figure 1A–C). There is no significant change of IgM titer against Omicron-RBD over two weeks (Figure 1A). Most interestingly, the titer of Wuhan-RBD- or Omicron-RBD-binding antibodies increased with the same kinetic (Figure 1B). In the first three days after the third dose, no increase in antibody titers was observed. An total of 50% of the maximum antibody titer is achieved for IgG after 5.1 d (Omicron 5.4 d), for IgM after 5.2 d (n.d. for Omicron), and for IgA after 5.3 d (Omicron 5.1 d). 99% of the maximum antibody titer is reached after 7.9 d for IgG (both variants), for IgA after 7.8 d (Omicron 7.1 d) and after 7.1 d for IgM (Omicron n.d.). Titers for IgA and IgG peak on day 9, but decrease by 42.3% for IgA (Omicron 66.6%) and 15.7% for IgG (Omicron 30.6%) by day 14.

The data described above indicate that at least three vaccinations are required to induce significant titers of Omicron-specific antibodies. This was confirmed by data from a different cohort (healthcare worker from a local hospital; Cohort 1; Figure 1D–E). A comparison of the titer of RBD-binding antibodies in sera obtained two weeks after the second vaccination to sera obtained about 10 weeks after the third vaccination revealed a moderate increase in the titer for the Wuhan-RBD and Delta-RBD-binding antibodies. In contrast to this, almost no Omicron-RBD-binding antibodies were observed after the second vaccination. The third dose caused an increase in the number of Omicron-RBD-binding antibodies (Figure 1D and Figure S1 in the before-after illustration). A comparison of the GMTs revealed that after the third vaccination, the GMTs for Wuhan-RBD- and Delta-RBD-binding antibodies are comparable, but the titer of the Omicron-RBD-binding antibodies is about 6.7 times lower than the titer of the Wuhan-RBD-binding antibodies. This is reflected by the titers determined in neutralization assays using an infectious virus. For the Delta variant, a PRNT50 geometric mean was determined as 201 for Omicron as 91 (Figure 1E).

This indicates that after the third dose, (i) titers of Omicron-RBD-binding antibodies are lower than the titers of Wuhan-RBD-binding antibodies, (ii) the increase in Wuhan-RBD- and Omicron-RBD-binding antibodies occurs with the same kinetic. As 99% of the maximum titer is reached 8 d after, people who received a third vaccination can be considered as completely boosted at this time point.

### 3.2. Qualitative Difference between Wuhan-Spike and Omicron-Spike Binding Antibodies in Sera of Three-Times Vaccinated Individuals

The next set of experiments was performed to characterize the immune response five to six month after the third dose. The ELISA revealed for the Wuhan-RBD and Delta-RBD-binding antibodies still robust GMTs, whereas the GMT for Omicron-RBD-binding antibodies was 4.5- and 4.0-fold lower, respectively (Figure 2A). In accordance with this, the analysis of the neutralizing activity by determination of the PRNT50 revealed that all sera preserved the capacity to neutralize the Delta variant. However, in the case of the Omicron variant, 35.9% of the analyzed sera failed to neutralize the virus, although for all sera Omicron-RBD-binding antibodies were determined (Figure 2B).

Apart from the titer, the stability of the antigen–antibody complex is a factor affecting the neutralizing capacity of sera. SPR analyses on a Biacore system were used to analyze the slow fraction, which describes the high-affinity fraction of the antibodies. The analyses showed that 5–6 months after the first booster vaccination, the stability of Delta-RBD antibody complexes was not affected compared with Wuhan-RBD antibody complexes. The stability of the antibody–Omicron-RBD complex is significantly smaller than that of the Wuhan and the Delta variants (Figure 2C).

These data indicate that the capacity of the sera to neutralize an infection by the Omicron variant five to six months after a third BNT162b2 dose is strongly reduced or no longer existent. Moreover, the fraction of antibodies binding with high affinity to Omicron-RBD is significantly smaller than the fractions binding to Wuhan-RBD or Delta-RBD.

### 3.3. Impact of a Fourth Vaccination on Antibody Titer and Stability of the Antigen–Antibody Complexes

The data described above reveal a waning of the immunity even after a booster vaccination. This is much more pronounced in the case of antibodies binding and neutralizing the Omicron variant than in antibodies specific for the ancestral isolate or the Delta variant. In light of the waning immunity, a second booster is recommended for elderly and certain groups at risk, such as healthcare workers [12,13].

To study the impact of a second booster vaccination (i.e., fourth vaccination) sera, which were taken five to six months after the third vaccination were compared to sera taken two weeks after the fourth vaccination (Figure 3A). ELISAs for the quantification of antibodies binding to the Wuhan-RBD, Delta-RBD or Omicron-RBD revealed that after the second booster vaccination, the GMT for the Wuhan-RBD- and Delta-RBD-specific antibody is elevated compared to the titer before the fourth vaccination (second boost). Moreover, the titer of Omicron-RBD-binding antibodies is 2.9-fold increased by the second booster vaccination (Figure 3A). Interestingly, although the titer of Omicron-RBD-specific antibodies was more than twofold increased by the second booster, the GMT was still 4.4-fold lower than the Wuhan-RBD- specific titer and 2.4-fold lower than the Delta-RBD-specific titer.

As described above, the analysis of the neutralizing titers revealed that a significant fraction of the sera collected more than 5 months after the third vaccination failed to neutralize infection by the Omicron variant (Figure 3B), although all sera at this time point still displayed a detectable level of Omicron-RBD-specific antibodies (Figure 3A). After the second booster vaccination, there was a significantly increased neutralizing capacity for the Delta variant and for the Omicron variant. At this time point, all sera had the capacity to neutralize the Omicron variant (Figure 3B).

Apart from the increase in the titer of RBD-binding antibodies, there is a qualitative change affecting the stability of the antigen–antibody complex, as revealed by analysis of the stability of the antigen–antibody complexes. This was studied by determination of the half-life of the antigen–antibody complexes by SPR. The stability of the antigen–antibody complex in the case of the Omicron variant is at all analyzed time points significantly lower than Wuhan-RBD and Delta-RBD-binding antibodies (Figure 3C). Interestingly, an increase in stable complexes between the second and third dose was observed with the Delta variant. However, after the first and second booster vaccination, the half-life of the antigen–antibody complex with the Omicron-RBD slightly increases (Figure 3D).

Taken together, these data indicate that a second booster vaccination induces a further increase in the antibody titer in the case of the Omicron-RBD-binding antibodies and a qualitative change, as reflected by an increase in the fraction representing antibodies with higher affinity.

### 3.4. Impact of the Vaccination Regimen on Antibody Titer and Specificity 

**Figure 4:**
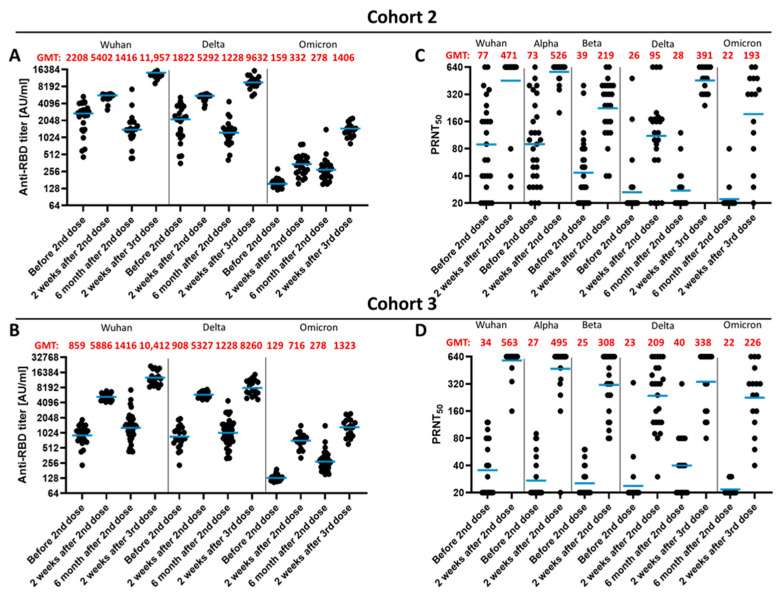
Comparison of different vaccination strategies. Sera from AZD1222/BNT162b2/BNT162b2-vaccinated and BNT162b2/BNT162b2/BNT162b2-vaccinated individuals from different time points were analyzed by ELISA and Plaque assay. (**A**,**B**) Sera (**A**, BNT162b2/BNT162b2/BNT162b2; **B**, AZD1222/BNT162b2/BNT162b2) were tested by ELISA using the RBD domain from different SARS-CoV-2 variants (Delta or Omicron, Wuhan, China) as antigen. AU/mL; Arbitrary unit per milliliter. (**C**,**D**) Plaque Assay with different SARS-CoV-2 variants. Plaque reduction neutralization titer against the SARS-CoV-2 virus, Alpha, Beta, Delta and the Omicron variant. Blue bar represents the geometric mean, GMT; geometric mean titer.

The next set of experiments was performed to investigate if heterologous and homologous vaccinations differ with respect to the waning of the immunity and to the effect of a third dose vaccination on the titers of Wuhan-RBD-, Delta-RBD- and Omicron-RBD-binding antibodies. For this purpose, sera was collected two weeks and six months after the second vaccination and two weeks after the third vaccination from individuals with a homologous vaccination (3 × BNT162b2) or with a heterologous vaccination (AZD1222/BNT162b2/BNT162b2). The sera were analyzed by ELISA for quantification of the Wuhan-RBD, Delta-RBD and Omicron-RBD-binding antibodies (Figure 4A,B). There are only marginal differences for the number of binding antibodies between the two vaccination regimens. Over time, there is a decrease in the Wuhan-RBD and Delta-RBD-binding antibodies, but there is still a robust titer detectable six months after the second vaccination. In the case of the Omicron-RBD, nearly no specifically binding antibodies were detectable before the third vaccination in the homologous vaccinated individuals. After the third vaccination, a comparable increase in the titer of Omicron-RBD-specific antibodies, which is significantly lower than the titer of Wuhan-RBD or Delta-RBD-specific antibodies, was observed. The vaccination regimen had no impact on these parameters (Figure 4A,B).

Analysis of the neutralizing capacity revealed that two weeks after the second vaccination, the titer of antibodies neutralizing the Beta or Delta variant is higher in the case of the sera derived from individuals with a heterologous vaccination than the homologous (AZD1222/BNT162b2/BNT162b2) vaccination. Six months after the second vaccination, the capacity to neutralize an infection by the Delta variant vanished in the case of the sera derived from BNT162b2/BNT162b2- (Figure 4C) vaccinated individuals, while 44% of the sera derived from the AZD1222/BNT162b2- (Figure 4D) vaccinated individuals still preserved a neutralizing activity of the Delta variant. Here, two sera from each group were able to neutralize with a low titer the Omicron variant. Sera obtained two weeks after the third vaccination revealed for the heterologous vaccination a slightly higher neutralizing capacity of the Delta variant than the homologous vaccination group. For both groups, a neutralizing effect on the Omicron variant was found, which is slightly higher in the case of the 3 × BNT162b2 group than in the AZD1222/BNT162b2/BNT162b2 group. However, compared to the Delta variant, the neutralizing titer for the Omicron variant is low (Figure 4C,D).

Taken together, these data indicate that the vaccination regimen (heterologous versus homologous vaccination) has no significant impact on the titer of Omicron-specific antibodies.

## 4. Discussion

Real-world data revealed for the mRNA vaccines (mRNA-1273 and BNT162b2) a very high vaccine effectiveness of more than 90% with respect to the prevention of COVID-19 [14,15]. Later on, data from Israel and from New York showed that there is a waning of the immunity [3,16]. The data from Israel demonstrated that especially in people over 60 years old, there is a significant decrease in vaccine effectiveness over time. A study from New York obtained data supporting the observation that over time, the SARS-CoV-2-specific immunity wanes. However, the data obtained from the study published by Bian et al. and a clinical trial by Qu et al. show that, in addition to time since completion of the vaccination, the emergence of variants has a significant impact on the decrease in vaccine effectiveness. In the abovementioned study (NCT05244330), the emergence of the Delta variant had a significant impact on vaccine effectiveness [17].

Both factors underline the need for a booster vaccination. The spike protein of the ancestral isolate (Wuhan) and of the Alpha, Beta or Delta variants differ only in a limited number of amino acids (aa), ensuring that a variety of neutralizing epitopes are preserved. In contrast to this, the antigenic distance of the Omicron variant is much more pronounced. In case of the Omicron variant, 37 aa are changed, affecting a variety of neutralizing epitopes, which has a strong impact on the titer of Omicron-binding antibodies after twofold vaccination with the licensed vaccines [18].

In light of this, booster vaccinations were performed. The success of a booster vaccination depends on the presence of memory B cells, which can be restimulated and contribute to a restoration of neutralizing antibody titers. Indeed, our data show that waning immunity, in the case of Wuhan-, Alpha- or Delta-RBD-specific antibodies, can be restimulated by a first booster vaccination (third vaccination) or even second booster (fourth vaccination).

Pseudotyped particles with a designed spike protein that differs in 25 positions from the Wuhan-spike protein were not neutralized by sera obtained from individuals vaccinated twice or by convalescent sera [9]. Further experiments revealed that sera from individuals with a breakthrough infection after two vaccinations or after a third vaccination developed neutralizing antibodies.

After two vaccinations with the mRNA vaccines, there are very low levels of Omicron-RBD-binding antibodies. Over time, after the third and fourth vaccination, there is an increase in titer of Omicron-RBD-binding antibodies and an increase in the neutralizing activity. While the restauration of the Wuhan-RBD-specific antibodies can be considered as a restimulation of the immune response, the emergence of Omicron-specific antibodies depends on a different mechanism. As observed in the abovementioned study using pseudotyped particles with about 25 mutations in the spike, a broadening of the immune response due to somatic hypermutation might be causative. However, the titers of Wuhan-specific antibodies generated by restimulation are significantly higher than the titers of the antibodies, which evolve over time by somatic hypermutation. The Delta-spike displays much more similarity to the Wuhan-spike than the Omicron-spike. Therefore, there is a robust Delta-RBD-specific titer induced already after two vaccinations. However, somatic hypermutation could contribute to the selection of antibodies binding to mutated epitopes in Delta.

Although different mechanisms lead to the formation of Wuhan-spike and Omicron-spike-specific antibodies, it was interesting to observe that after the first booster vaccination, the formation of these antibodies occurs with the same kinetic, although there is a clear difference in the titer between Wuhan-spike and Omicron-spike-specific antibodies. The Wuhan-spike-specific titer is about 7.3-fold higher. The data show that for IgG, the maximum level is reached 7 d after the first booster vaccination.

In contrast to this, the waning occurs with different kinetics. In case of the Omicron-specific sera six months after the second vaccination, 72.2% of the sera failed to neutralize an infection by the Omicron variant. Apart from the waning, the initial lower titer and the higher infectivity of the Omicron variant might contribute to this. However, it is interesting that five to six months after the third vaccination, there is still a titer of Omicron-RBD-binding antibodies in all analyzed samples detectable, but 35.9% fail to neutralize an Omicron infection. This reflects a qualitative difference between the antibodies. While in the case of Wuhan-RBD-binding antibodies, the stability of antigen–antibody complexes is significantly higher than the stability of the Omicron-RBD-binding antibodies.

## 5. Conclusions

These data indicate that even a repetitive booster vaccination based on the Wuhan isolate has a limited capacity to induce a long-lasting humoral immune response against a distant variant such as Omicron. The low titer of Omicron-specific antibodies and the reduced stability of the antigen–antibody complexes might be relevant factors, which might be causative for the reduced neutralization of Omicron by sera raised due to immunization with the Wuhan-spike protein. This indicates the urgent need for the development of a variant-adapted second generation of SARS-CoV-2 vaccines.

## Figures and Tables

**Table 1:** Recruited cohorts within this study.

Cohort	Vaccination Schedule	Serum Collection	Samples	Age *	Sex *
**1**	D1 = 0 weeks; BNT162b2 D2 = 3 weeks; BNT162b2 D3 = 6 months; BNT162b2	T1 = 2 weeks after second dose	*n* = 20	M = 46; (20–64)	m = 12; f = 9
		T2 = 10 weeks after third dose	*n = 20*	M = 46; (20–64)	m = 12; f = 9
**2**	D1 = 0 weeks; BNT162b2 D2 = 3 weeks; BNT162b2 D3 = 6 months; BNT162b2	T1 = 3 weeks after first dose (before second dose)	*n* = 26	M = 43; (22–66)	m = 12; f = 14
		T2 = 2 weeks after second dose	*n = 23*	M = 43; (22–66)	m = 11; f = 12
		T3 = 6 months after second dose (before third dose)	*n = 18*	M = 43; (22–64)	m = 5; f = 13
		T4 = 2 weeks after third dose	*n = 15*	M = 40; (22–60)	m = 3; f = 12
**3**	D1 = 0 weeks; AZD1222 D3 = 8 weeks; BNT162b2 D4 = 6 months; BNT162b2	T1 = 8 weeks after first dose (before second dose)	*n* = 24	M = 50; (26–63)	m = 11; f = 13
		T2 = 2 weeks after second dose	*n = 24*	M = 49; (26–63)	m = 10; f = 14
		T3 = 6 months after second dose (before third dose)	*n = 20*	M = 50; (26–63)	m = 10; f = 10
		T4 = 2 weeks after third dose	*n = 16*	M = 51; (26–63)	m = 8; f = 8
**4**	D1 = 0 weeks; AZD1222 D2 = 3 weeks; BNT162b2 D3 = 6 months; BNT162b2 D4 = 12 months; mRNA-1273	T1 = 6 months after third dose (before fourth dose)	*n* = 36	M = 47; (25–65)	m = 12; f = 24
		T2 = 2 weeks after fourth dose	*n = 19*	M = 46; (25–63)	m = 7; f = 12
**5**	D1 = 0 weeks; BNT162b2 D2 = 3 weeks; BNT162b2 D3 = 6 months; BNT162b2	T1 = One day before third vaccination	*n* = 6	M = 28; (25–33)	m = 1; f = 5
		T2-15 = Daily	*n = 6*	M = 28; (25–33)	m = 1; f = 5

* M: Mean; range of the age is given in brackets; m: male; f: female.

## Data Availability

All supporting data are included in this manuscript.

## Supplementary Materials

The following supporting information can be downloaded at: https://www.mdpi.com/article/10.3390/vaccines10050796/s1, Figure S1: ELISAs of the four cohorts in the before-after graph.
