# Supplementary material for: Quantitative and Qualitative Difference in Antibody Response against Omicron and Ancestral SARS-CoV-2 after Third and Fourth Vaccination
Source: Vaccines (Basel). 2022 May 17;10(5):796. doi: 10.3390/vaccines10050796 (PMC9145667; doi:10.3390/vaccines10050796)
Supplement: Supplementary file 1 [file vaccines-10-00796-s001.zip › vaccines-1712098-supplementary.pdf]

Supplementary: Quantitative and qualitative difference in antibody response against omicron and ancestral SARS-CoV-2 after third and fourth vaccination

Sascha Hein [1], Ines Mhedhbi [1], Tobias Zahn [1], Catarina Sabino [1], Nuka Ivalu Benz [1], Younes Husria [1], Patricia Maria Renelt [1], Floriane Claudia Maria Braun [1], Doris Oberle [2], Thorsten Jürgen Maier [2], Christoph Hildt [3], Eberhard Hildt [1,*]

AFFILIATION:

1 Paul-Ehrlich-Institut, Department of Virology, Paul-Ehrlich Street 51-59, D-63225 Langen, Germany

2 Paul-Ehrlich-Institut, Safety of Medicinal Products and Medical Devices Division, Paul-Ehrlich Street 51-59, D-63325 Langen, Germany

3 Main-Kinzig-Kliniken, Herzbachweg 14, D-63571 Gelnhausen, Germany 10, D-13353 Gelnhausen, Germany

* Correspondence: Eberhard.Hildt@pei.de

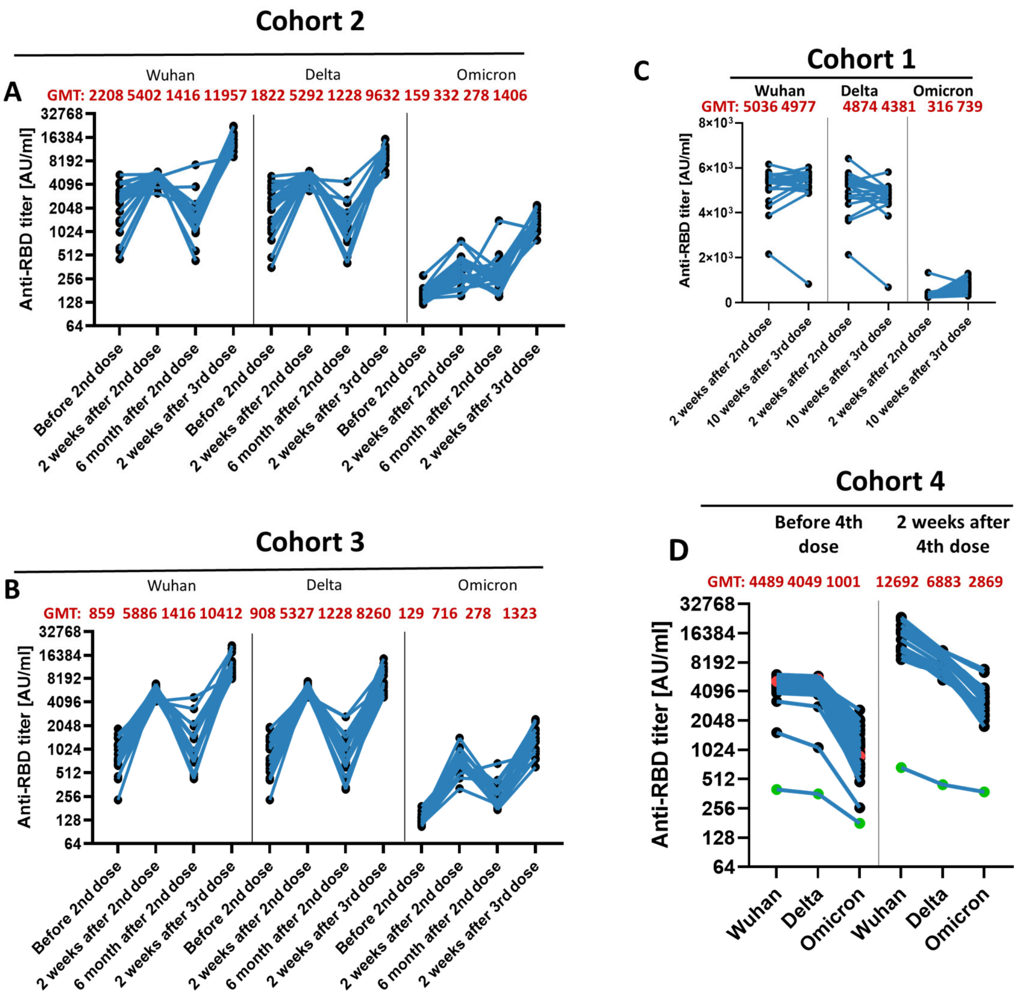

Figure S1 ELISAs of the four cohorts in the before-after graph. It can be clearly seen that all sera show a similar pattern. Sera were tested by ELISA using the RBD domain from different SARS-CoV-2 variants (Wuhan, delta or omicron) as antigen. AU/ml; Arbitrary unit per milliliter. Red dots: Patients became infected with omicron variant 3 days after blood collection. Green: Patient is immunosuppressed. GMT; geometric mean titer.
